# Primary Health Care Utilization by the Mexican Indigenous Population: The Role of the *Seguro Popular* in Socially Inequitable Contexts

**DOI:** 10.1371/journal.pone.0102781

**Published:** 2014-08-06

**Authors:** Rene Leyva-Flores, Edson Servan-Mori, Cesar Infante-Xibille, Blanca Estela Pelcastre-Villafuerte, Tonatiuh Gonzalez

**Affiliations:** Centre for Health Systems Research of the National Institute of Public Health, Cuernavaca, Morelos, Mexico; Public Health Agency of Barcelona, Spain

## Abstract

**Objective:**

To analyze the relationship between primary health care utilization and extended health insurance coverage under the Seguro Popular (SP) among Mexican indigenous people.

**Methodology:**

A cross-sectional analysis was conducted using data from the Mexican National Nutrition Survey 2012 (n = 194,758). Quasi-experimental matching methods and nonlinear regression probit models were used to estimate the influence of SP on primary health care utilization.

**Results:**

25% of the Mexican population reported having no health insurance coverage, while 59% of indigenous versus 35% of non-indigenous reported having SP coverage. Health problems were reported by 13.9% of indigenous vs. 10.5% of non-indigenous; of these, 52.8% and 57.7% respectively, received primary health care (p<0.05). Economic barriers were the most frequent reasons for not using primary health care services. The probability of utilizing primary health care services was 11.5 percentage points higher (p<0.01) for indigenous SP affiliates in comparison with non-indigenous, in similar socioeconomic conditions.

**Conclusion:**

Socioeconomic conditions, not ethnicity per-se, determine whether people utilize primary health care services. Therefore, SP can be conceived as a public policy strategy which acts as a social *buffer* by enhancing health care utilization regardless of ethnicity. Further analysis is required to explore the potential gaps as a result of SP coverage among socially vulnerable groups.

## Introduction

Worldwide, access to health care for indigenous groups has always been limited due to social inequity [1]–[3]. Although representing 5% of the world's population, indigenous peoples account for approximately 15% of global poverty [4] and one-third of extreme poverty in rural areas [4].

Mexican indigenous people (∼7% of the Mexican population in 2010) [5] are characterized by steep marginalization levels and wide social gaps [6], [7]. In 2010, 44.2% of those residing in indigenous municipalities [8] suffered from extreme poverty (according to the Mexican National Council for the Evaluation of Social Development Policy or CONEVAL, extreme poverty is a condition characterized by such meager incomes that, even when destined entirely to purchasing food, they prove insufficient for acquiring the necessary nutrients to lead a healthy life); 78.6% earned less than three minimum wages; 83.3% were below the well-being threshold or under an income equivalent to the total value of the consumption food and non-food bundle per person per month (according to CONEVAL at April 2013: US$122.1 in localities with less than 2500 inhabitants or rural and US$190.3 and localities with more than 2,500 inhabitants or rural), and 75.6% lived in marginalized communities [6]. Additionally, in 2010, the infant mortality rate was 63% higher among indigenous versus non-indigenous municipalities, and prevalence of stunting was threefold in indigenous in comparison with non-indigenous communities [9]. These figures relate directly to the living conditions of indigenous peoples and the countless obstacles they face in accessing primary health care services.

The Mexican government has responded by stepping up social policies that seek greater access to education, food and health [10], particularly for populations stricken by acute social vulnerability, such as the indigenous communities.

Since 2003, Seguro Popular (SP) has constituted one of several social health protection strategies created to remove economic barriers and improve access to health care [11], with the SP program providing public funds for populations in the first income deciles who lack social security [11]–[14]. Since its inception, public resources allocated to SP have grown 13.3 times, that is, from $382.6 million USD in 2004 to $5,087.6 million USD in 2012 ($4,315.3 million MXN-2004- vs. $67,004 million MXN-2012, according to BANXICO, average monthly peso/dollar exchange rates were 11.28 in 2004 and 13.17 in 2012), when the annual average reached $100 USD per affiliate [15].

Seguro Popular impact assessment studies have demonstrated its protective effect against catastrophic health spending among its beneficiaries [12]–[14], [16]–[18], and analyses on health care utilization have sighted a number of contributions [19], [20]. However, evidence regarding performance in rural indigenous communities at the lowest economic levels [21], [22] suggests that results have been less favorable among these beneficiaries. *SP* coverage increased from 14% to 61.9% for the indigenous population from 2006 to 2012, but from 10% to 35.7% for the non-indigenous population throughout the ten years following its implementation [23]. In order to establish the relationship between primary health care utilization and extended insurance coverage for Mexico's poorest, specifically for the indigenous population, this study analyzed the role of *SP* as a socioeconomic *buffer* against barriers to health care services in 2012.

## Methodology

A cross-sectional study was conducted with data from the Mexican National Nutrition Survey (ENSANUT) 2012, a probabilistic survey providing evidence not only at national and state levels, but also by urban and rural strata [24]. Data from the survey's household module were retrieved specifically with regard to socio-demographic indicators, health status and primary health care utilization. This survey was approved by the Research and Ethics Committees of the National Institute of Public Health. ENSANUT was applied to 194,758 persons in 50,528 households, excluding individuals who provided incomplete socio-demographic data (0.74%); already benefited from private health insurance (0.34%), or lacked information on morbidity, type of health problem, or attendance at primary health care services (0.4%). With a sampling loss of 1.5%, analyses were performed on a final sample of 191,849 individuals (N = 113,039,438).

The variable of interest was primary health care utilization. This is an indicator that has proved sensitive to shifts in economic capacities influencing the regulation of health care demand [25]–[27]. Congruent with previous studies, primary health care utilization was defined according to the respondents' self-reported health problems - both experienced within two weeks prior to ENSANUT, and treated by medical personnel on an outpatient basis [28]. People who reported being attended to by non-medical personnel (13.9%) were classified as non-users.

The indigenous population was identified according to the definition proposed by the National Commission for the Development of Indigenous Peoples (CDI for its initials in Spanish), whereby a household is considered indigenous if the head of the family, a spouse and/or an ascendant self-identifies as a speaker of an indigenous language [29]. 24,090 people (∼10.8 million individuals, or 9.6% of the total Mexican population) were thus identified as indigenous.

Analyses were performed on individual and community-related factors linked by former studies [28], [30]–[34] to the likelihood of receiving health care and/or utilizing health services. Individual variables included: gender, age, education, employment, marital status, number of household members, annual spending (per resident per capita) and receipt of cash transfers from the Mexican Oportunidades anti-poverty program. Places of residence included locality sizes: (i) rural (<2,500 inhab.); (ii) urban (2,500–100,000 inhab.), and (iii) metropolitan (>100,000 inhab.). Lastly, marginalization levels were classified as: (i) very low/low; (ii) medium, and (iii) high/very high [35].

In terms of processed data, the study began by describing the socio-demographic characteristics of the indigenous/non-indigenous groups. Statistically different group traits were obtained by applying statistical independence tests (χ^2^ for categorical variables and t-student for continuous variables). Additionally, health assurance categories were determined as: (i) having Social Security (SS), (ii) having SP, and (iii) having no SS or insurance whatsoever.

The SS population was then excluded from the study sample to achieve more homogeneous groups, given that 86% of the indigenous population reported having no SS. At this point, the analytical sample was circumscribed exclusively to SP versus non-SP subjects (63% of the total population). Analyses were then performed on primary health care utilization for the self-reported health problems mentioned above (defined as chronic, acute or others) [33]. In the case of problems unattended to by health personnel, impediments cited by respondents were classified as: (i) factors related to service demand (lack of money); (ii) factors related to service supply (ie. lack of confidence, ill-treatment, unavailability and remoteness), and (iii) other causes. Results were then estimated for the sample population, bearing in mind the impact of design on the survey.

Assessing the impact of being indigenous on the likelihood of obtaining primary health care was subject to confounding factors and potential differences inherent in a self-selected study sample marked by the nonrandom assignment of indigenous subjects and the non-experimental design of ENSANUT. Propensity score matching (PSM) was thus applied, as it is one of the methods most frequently used for recreating experimental conditions and obtaining causal inference. PSM allows constructing an appropriate comparison group vis-à-vis the treatment group, as well as controlling for biases indicated by observable variables [36]–[39].

The indigenous/non-indigenous populations were thus matched according to the co-variables described above, using the one-to-one nearest neighbor algorithm [39]. This and further analyses on matching confirmed statistical similarity between the two groups and bias correction. Non-linear probability probit models [40] were then applied to the matched sample to assess how the attainment of primary health care from medical personnel (adjusted by PSM) was influenced, not only by ethnicity, but also by the interaction of ethnicity with SP affiliation. Marginal effects (in percentage points or pp) and 95% confidence intervals were obtained. All analyses were carried out with STATA SE v13.1 software [41].

## Results

Table 1 illustrates the socio-demographic characteristics of the indigenous/non-indigenous study populations. While men and women were distributed proportionately between the groups, indigenous respondents belonged to a younger age bracket (0–19 yrs., 45.3%) than their non-indigenous counterparts (20–49 yrs., 43.3%), and presented lower education levels, participation in the labor market, and per capita spending (∼$68.1 USD/month *vs*. $128.9 USD/month). Additionally, significantly more indigenous (44.1%) than non-indigenous (14.1%) respondents occupied Quintile I of per capita spending, belonged to *Oportunidades* households (58.2% *vs.* 22%), and dwelled in both rural localities (53.5% *vs*. 19.4%) and highly marginalized areas (71.3% *vs*. 17%).

**Table 1:** Socio-demographic conditions of the indigenous and non-indigenous Mexican population, 2012.

	Non-indigenous	Indigenous	Differences in means or percentages p value
n	168,263	24,163	
N	102,600,000	10,855,299	
%	90.4	9.57	
**Socio-demographic characteristics**			
Men	48.9 [48.6,49.1]	49.0 [48.2,49.7]	0.85
Age group			
0–4	9.45 [9.24,9.66]	10.5 [9.88,11.0]	**0.00**
5–19	29.5 [29.1,29.8]	34.8 [33.7,35.9]	
20–49	43.3 [43.0,43.6]	38.0 [37.1,38.8]	
50–69	13.8 [13.4,14.1]	12.7 [12.0,13.4]	
Over 69	4.05 [3.84,4.27]	4.15 [3.71,4.64]	
Education (yrs.)*			
0	9.56 [9.32,9.81]	19.7 [18.6,20.7]	**0.00** *
1–6	30.7 [30.2,31.2]	38.4 [37.2,39.5]	
7–9	24.0 [23.6,24.3]	18.6 [17.7,19.5]	
10–12	16.1 [15.7,16.5]	9.04 [8.26,9.88]	
13 or more	10.2 [9.69,10.8]	3.96 [3.40,4.61]	
Not applicable: < 5 years of age	9.45 [9.24,9.66]	10.5 [9.88,11.0]	
Employed*			
No	47.6 [47.2,48.0]	51.5 [50.6,52.3]	**0.00** *
Yes	37.1 [36.7,37.5]	31.4 [30.4,32.4]	
Not applicable: < 8 years of age	15.3 [15.0,15.6]	17.2 [16.4,18.0]	
Marital status*			
Married or living together	41.3 [41.0,41.6]	39.9 [39.1,40.8]	**0.00** *
Divorced	4.11 [3.96,4.26]	2.14 [1.86,2.47]	
Widow(er)	3.31 [3.18,3.45]	3.18 [2.89,3.49]	
Single	28.0 [27.6,28.4]	27.7 [26.7,28.7]	
Not applicable: <12 years of age	23.4 [23.0,23.7]	27.1 [26.0,28.1]	
**Household**			
Receives Oportunidades transfers	22.0 [21.2,22.8]	58.2 [55.4,61.0]	0.00
Total number of members	4.73 [4.69,4.78]	5.41 [5.29,5.53]	**0.00**
Annual spending per capita (MXN 000)	19.7 [19.2,20.2]	10.4 [9.79,10.9]	**0.00**
Annual spending per capita (quintiles)			
I	14.1 [13.3,14.9]	44.1 [40.8,47.5]	**0.00**
II	17.8 [17.2,18.4]	21.0 [19.2,22.9]	
III	20.2 [19.5,20.9]	15.4 [13.8,17.3]	
IV	22.5 [21.9,23.2]	11.6 [10.2,13.1]	
V	25.5 [24.5,26.5]	7.93 [6.78,9.25]	
**Locality or municipality of residence**			
Rural (<2500 inhab.)	19.4 [18.6,20.3]	53.5 [48.2,58.7]	**0.00**
Urban (2500–100 thousand inhab.)	18.6 [18.0,19.3]	22.9 [18.6,27.9]	
Metropolitan (>100 thousand inhab.)	61.9 [60.9,62.9]	23.6 [19.7,28.1]	
Level of marginalization			
Very low/low	71.7 [70.0,73.3]	21.2 [18.1,24.6]	**0.00**
Medium	11.3 [9.90,13.0]	7.53 [5.08,11.0]	
High/very high	17.0 [15.6,18.4]	71.3 [66.8,75.4]	

Source: Mexican National Nutrition Survey 2012.

Note: Estimates take into account the effect of survey design.

*p value calculation excludes non-applicable categories.

On comparing indigenous with non-indigenous health insurance enrollment (Table 2), the former proved lower (2.8 times) with *SS,* but higher with *SP* (58.7% and 34.5%, respectively). Furthermore, 27% of the indigenous and 25.5% of the non-indigenous study population reported having no health insurance whatsoever.

**Table 2:** Health Insurance among indigenous and non-indigenous Mexican population, 2012.

	Non-indigenous	Indigenous	Differences in percentages p value
n	168,263	24,163	
N	102,600,000	10,855,299	
%	90.4	9.57	
Individual enrollment institution			
Social security	40.1 [39.0,41.1]	14.2 [12.6,16.0]	**0.00**
Seguro Popular health insurance	34.5 [33.6,35.4]	58.7 [55.9,61.5]	
None	25.5 [24.8,26.2]	27.0 [24.9,29.3]	

Source: Mexican National Nutrition Survey, 2012.

Note: Estimates take into account the effect of survey design.

**Figure 1:**
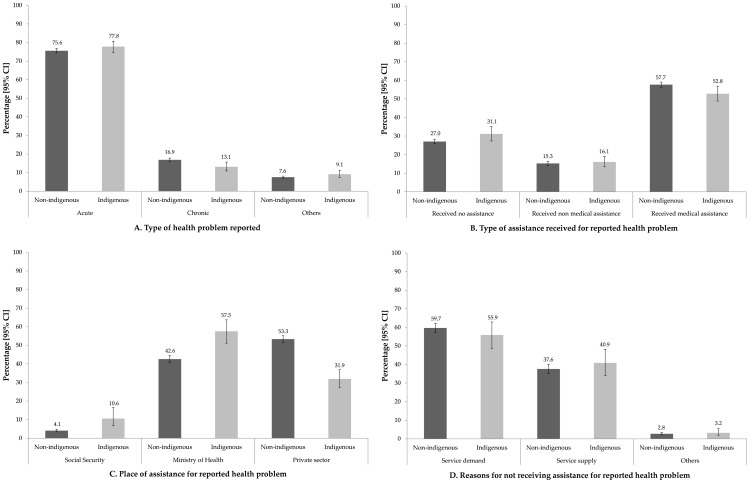
Indigenous population: morbidity, type and place of healthcare, and reasons for not using primary health care services. A. Type of health problem. B. Type of assistance received for reported health problem. C. Place of assistance for reported health problem. D. Reasons for not receiving assistance for reported health problem. Source: Mexican National Nutrition Survey, 2012. Note: Estimates take into account the effect of survey design. Social security beneficiaries were excluded.

Of the total number of individuals affiliated (or not) to *SP* who reported health problems in the two weeks prior to *ENSANUT*, 13.9% were indigenous and 10.5% non-indigenous. Also, slight differences were observed with regard to health problems (Figure 1). Indigenous people suffered more from acute problems while their non-indigenous counterparts suffered more from chronic problems (Panel A). Additionally, fewer indigenous (52.8%) than non-indigenous subjects (57.7%) received any kind of health care (Panel B). Lastly, 10.6% of indigenous against 4.1% of non-indigenous attended *SS* facilities. 57.4% of indigenous and 42.6% of non-indigenous subjects received health care by the Ministry of Health (*SSA* according to its initials in Spanish) while 32% of indigenous and 53.3% of non-indigenous people utilized private physicians (Panel C). Regarding those who did not utilize primary health care services, 59.7% of non-indigenous and 55.9% of indigenous respondents cited reasons associated with lack of money as the main barrier. Other reasons referred to service supply (37.6%) and perception of services (40.9%), including lack of confidence, poor treatment, unavailability and remoteness. No significant differences were observed between the groups in this regard (Panel D).

Considering similar socioeconomic conditions, Table 3 shows the estimated impact of ethnicity on the probability of primary health care utilization. In general, Model 1 yielded a 5.69 pp higher probability for indigenous versus non-indigenous subjects. However, after including *SP* enrollment, Model 2 yielded the same probability for both populations, but a 10.2 pp higher probability for *SP* beneficiaries. Lastly, after adding the interaction between the two variables, Model 3 yielded an 11.5 pp higher probability of primary health care utilization for individuals who were both indigenous and *SP* affiliates. The variables proved statistically insignificant when analyzed independently.

**Table 3:** Impact of being indigenous on the probability of using primary health care services.

	Probit Model – Percentage points [95% CI] reported		
	(1)	(2)	(3)
Indigenous	**5.69 [2.38,9.00]** **	**5.73 [2.40,9.05]** **	−3.19 [−10.1,3.72]
Seguro Popular Health Insurance		**10.2 [6.25,14.1]** **	4.41 [−1.18,10.0]
Indigenous x Seguro Popular Health Insurance			**11.5 [3.78,19.3]** **
Observations	3,490	3,490	3,490

Source: Mexican National Nutrition Survey, 2012.

**p<0.01.

## Discussion

Social inequity has been a persistent problem among ethnic groups throughout the world [1]–[3]. Mexico, a country with an array of indigenous communities historically marked by deep social gaps, is not spared from this reality. To overcome social inequity and improve the living conditions of vulnerable groups, namely the indigenous communities [11], [42], the Mexican government has implemented a host of initiatives. For example, in the early 1940's there was an emergence of social development programs, some of which followed an “assistentialist” approach and have remained untouched in their content since then (see catalog of Federal Social Development Programs and Actions published by the CONEVAL). In 2011, only 14 (5.1%) out of 273 Federal Programs and Actions for Social Development were aimed specifically at the indigenous population, representing a scant 1.4% of total financial resources allocated to national social policy, or ∼0.1% of GDP (Federal Programs and Actions for Social Development were budgeted $730,581.47 million MXN). Moreover, although 40% of the 273 programs related to health care, none were targeted to the indigenous population (see Social Policies in Mexico: Progress and Challenges in 2012, CONEVAL).

Currently, SP is one of the most ambitious social programs undertaken by the Mexican government. It aims to provide financial protection regarding health for the poorest and non-Social Security insured. It also aims to provide opportune and quality access to medical, surgical, pharmaceutical and hospital services required to comprehensively meet the health needs of its beneficiaries [43]. This study focused on primary health care utilization by indigenous people, underscoring the role of SP in facilitating these services in acutely inequitable contexts. Based on quasi-experimental methods, our findings endorse the hypothesis that SP offsets the barriers preventing the use of primary health care services by indigenous and non-indigenous in similar socioeconomic conditions. Our findings suggest that it is not being indigenous per se, but rather the lack of financial assurance for accessing health care, that hinders primary health care utilization. In principle, from this perspective, SP can be conceived as a public policy strategy that acts as a buffer by enhancing primary health care utilization regardless of ethnicity. However, given the persistence of countless uninsured Mexicans [22], the SP social policy may be having an adverse effect by widening the gap between those who utilize and not health services in the poorest population segments. In other words, having or not SP benefits may actually be shaping a new social gap within the vulnerable population segments. Socio-political outlooks on this matter span the gradualist spectrum. Some outlooks have the hope that universal coverage will eventually occur and contribute to social equity in health, while public statements from recent governments [11] affirm that this goal has already been met. To the contrary, however, evidence published by Laurell [22], the National Council for the Evaluation of Social Development Policy (CONEVAL for its initials in Spanish) [12] and, more recently, by ENSANUT 2012, demonstrates that as many as 21.4% of the Mexican population do not benefit from any health insurance whatsoever [44].

While recognizing that “SP has achieved 51.8 million affiliates,” CONEVAL reports that “there is a population group that turns elsewhere for its health-related financial risk management” [12]. For instance, in the case of indigenous communities, one out of ten individuals is covered by the Mexican SS system, a mechanism for providing expanded services, that is, services unrestricted to a catalog such as the one established under SP financing.

What exactly is the contribution of the SP financial insurance plans to socially vulnerable groups? Studies have identified that their greatest contribution lies in the reduction of household catastrophic health spending [12], [13], although exclusively for the health problems listed under the General Health Services Catalog (CAUSES for its initials in Spanish) [22]. CONEVAL [12] and Laurell [22] have indicated the following shortcomings in SP's organization as being potentially responsible for the observed gaps in primary health care utilization: inadequate distribution of physicians, insufficient accreditation mechanisms to ensure quality care, long wait times, and limited access to information on the rights of beneficiaries. According to CONEVAL, the claims in the official SP publications differ from the public perception, particularly as regards effective access and service quality” [12].

Finally, this study did not intend (and was therefore not designed) to assess either the effect of SP on population health status or the impact of SP organization on health care utilization. Studies with a distinct focus on these variables are required to meet the growing interest in the results of government programs for social development and poverty reduction. The ensuing analyses and existing evidence on these programs would contribute to a deeper understanding of their scope, functionality and role in public well-being.
